# Disseminated Necrotizing Leukoencephalopathy Complicating Septic Shock in an Immunocompetent Patient

**DOI:** 10.1155/2017/1092537

**Published:** 2017-04-24

**Authors:** Pedro Gaspar-da-Costa, Sofia Reimão, Sandra Braz, João Meneses Santos, Rui M. M. Victorino

**Affiliations:** ^1^Department of Internal Medicine, Hospital de Santa Maria and Faculty of Medicine of Lisbon, Lisbon, Portugal; ^2^Department of Neurologic Imaging, Hospital de Santa Maria and Faculty of Medicine of Lisbon, Lisbon, Portugal; ^3^Faculty of Medicine of Lisbon, Lisbon, Portugal

## Abstract

Disseminated necrotizing leukoencephalopathy (DNL) is characterized by multiple microscopic foci of white matter necrosis. DNL was initially thought to be exclusively associated with immunosuppression conditions but it has been recently described in immunocompetent patients in septic shock. A 90-year-old immunocompetent woman with no previous neurological impairment presented with septic shock and drowsiness that responded well to therapy with clinical improvement and a full neurological recovery. Unexpectedly deterioration with progression to coma occurred. Investigation excluded other causes and Magnetic Resonance Imaging (MRI) was consistent with the diagnosis of DNL showing bilateral multifocal white matter lesions with a nonvascular pattern with restricted diffusion. Neurological impairment persisted with progression to death. DNL is an unexpected diagnosis in an immunocompetent patient. We compared the present case to those found in the literature of DNL complicating septic shock and discuss the antemortem diagnosis based on MRI findings.

## 1. Introduction

Disseminated necrotizing leukoencephalopathy (DNL), also known as multiple necrotizing leukoencephalopathy (MNL), is a clinical entity with a poorly understood pathobiology [1]. It was first described in 1975 [2] by Rubinstein et al. in four children with leukemia and one child with Burkitt's lymphoma with central nervous system involvement that were under treatment with high dose systemic and intrathecal chemotherapy. Since then DNL has been identified in all age groups and in other immunosuppression states, such as Acquired Immunodeficiency Syndrome (AIDS) [1–6].

DNL has also been named pontine leukoencephalopathy due to reports of preferential involvement of this central nervous system area [6, 7], although there is consistent evidence that most patients have disseminated and multifocal white matter lesions [4, 6]. It was initially described as a rare complication of these specific immunocompromised patients but recent studies identified a possible link with the state of immune deregulation in septic shock [1, 8]. Typical histopathologic findings are microscopic multifocal white matter foci of necrosis, oedema, axonal swelling, and demyelinization [6] with diverse clinical manifestations such as a pyramidal syndrome, cerebellar dysfunction, behaviour alterations, and coma [1–12]. No specific clinical signs or symptoms are associated with the disease and the brain involvement of leukemia or lymphoma and the frequent use of intravenous sedation in septic patients further confound the clinical manifestations of DNL [1, 8]. Although definite confirmation relies on histology, Magnetic Resonance Imaging (MRI) is fundamental for the diagnosis of DNL in view of the characteristic features of bilateral multifocal supra and infratentorial white matter lesions with a nonvascular distribution with restricted diffusion [3, 13].

The present case illustrates the rarely reported unexpected association between septic shock and DNL and the importance of the MRI in the diagnosis of this clinical entity with poor prognosis.

## 2. Case Report

A 90-year-old women with known diagnosis of hypertension and otherwise healthy presented with drowsiness, abdominal pain, nausea, vomiting, fever, tachycardia, and hypotension with a blood pressure of 88/62 mmHg. Heart, lung, and abdominal observation were unremarkable. Neurological examination showed no focal or meningeal signs. Laboratory tests revealed thrombocytopenia (85000/uL) and a significant increase in leukocytes (16860/uL), neutrophils (15800/uL), C-reactive protein (18.1 mg/dL), blood urea nitrogen (93 mg/dL), and creatinine (2.8 mg/dL). Urinalysis had significant leukocyturia (>500 leukocytes/uL) and arterial blood gas analysis showed hyperlactacidemia (38 mg/dL) and metabolic acidosis (HCO_3_^−^: 19.8 mmol/L). Her chest roentgenography and electrocardiogram had no significant changes and the renal ultrasound found pathologic alterations in the right kidney with slight pyelocaliceal dilation and perirenal fluid. A diagnosis of acute pyelonephritis was made and the laboratory and hemodynamic parameters were consistent with septic shock and multiple organ failure.

Blood and urine cultures were obtained and prompt broad spectrum antibiotic therapy was initiated. Concurrent fluid resuscitation and hemodynamic support were provided and after a transient 24-hour worsening period, a steady progressive improvement was noted.* Escherichia coli* was isolated from both blood and urine samples and antibiotic adequacy was confirmed. Over the following days, clinical improvement was evident with full neurological recovery to previous cognition status, absence of fever, nausea, and abdominal pain. Laboratory tests also had a favourable evolution with a recovery of kidney failure and haematological dysfunction. Significant decrease in the inflammatory markers was also noted.

The patient had no previously neurocognitive impairment and the initial drowsiness resolved alongside with the global improvement. Despite this initial response, on the 5th day of hospitalization, there was sudden neurological deterioration to coma. No meningeal or focal signs were apparent and there was no evidence of tonic or clonic movements. Examination also excluded neurologic or hemodynamic signs of intracranial hypertension. Laboratory reevaluation was unremarkable and metabolic causes were excluded, namely, hypoglycemia, dysnatremia, uraemia, and hyperammonemia. Iatrogenic pharmacological causes were also absent.

**Figure 1:**
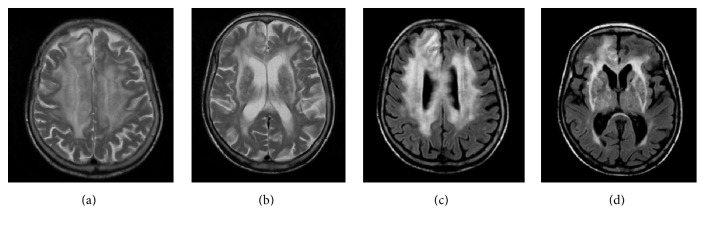
Axial T2 weighted (a, b) and FLAIR (c, d) MR imaging showing extensive white matter hyperintensity signal changes with a confluent pattern in periventricular and deep white matter of the cerebral hemispheres, without vascular distribution, more extensive on the right frontal region and involving all segments of the corpus callosum.

**Figure 2:**
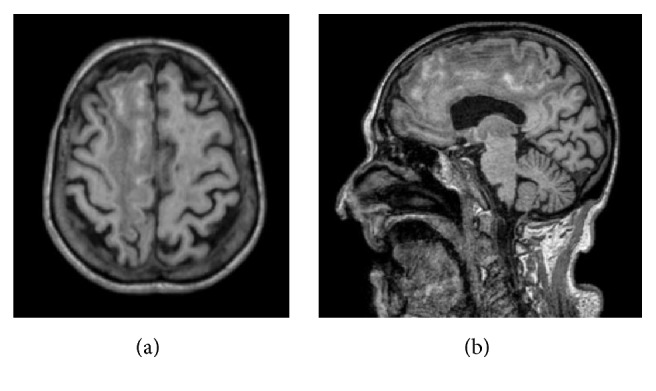
Axial (a) and sagittal (b) T1 weighted MR images. Linear spontaneous hyperintensity of the cortex in the frontoparietal convexity, compatible with laminar necrosis.

**Figure 3:**
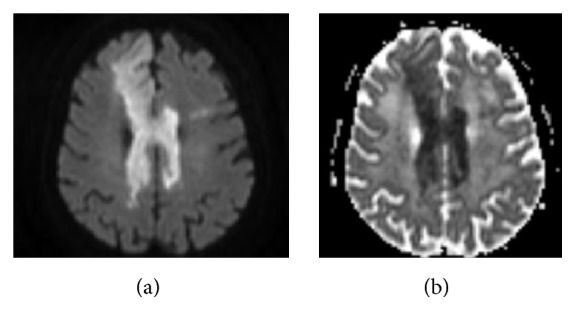
MR diffusion weighted images. DWI (a) and ADC map (b) with significant restricted diffusion of the corpus callosum bilaterally in all the segments and in the right anterior frontal and parietal region.

**Figure 4:**
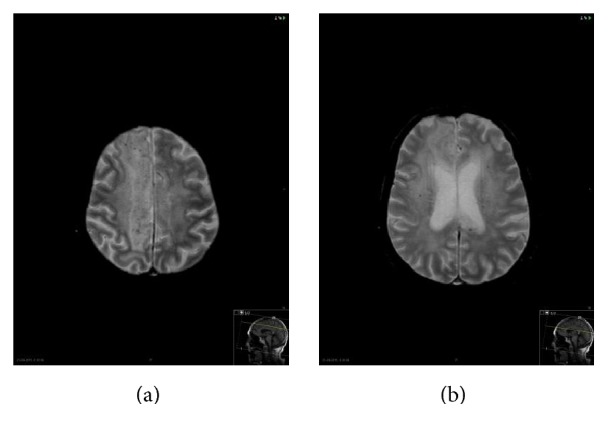
Axial T2^*∗*^. Punctate hypointensity foci of magnetic susceptibility diffusely in the central and subcortical cerebral white matter, without vascular distribution, corresponding to hemosiderin deposition.

This sudden neurological deterioration was unexpected and thus a diagnostic approach for other possible causes such as vascular, epileptic, and septic encephalopathy was done. Brain Computerized Tomography (CT) scan revealed a possible ischemic cortical and subcortical right frontal lesion with attenuation of the adjacent sulci. There were no haemorrhages, hydrocephalus, or midline shifts. As this possible ischemic lesion did not explain the neurological status, an electroencephalography was performed and showed slow bilateral frontal and temporal activity with periodical 1 to 2 Hz discharges. This was not diagnostic of status epilepticus and was suggestive of a low epileptic threshold. Neurologic and neurophysiologic specialist consultation was obtained and valproic acid was initiated, without neurologic benefit. A brain Magnetic Resonance Imaging (MRI) was then performed and showed bilateral supra and infratentorial multifocal white matter lesions, with a pattern that did not follow vascular territorial distribution (Figures 1, 2, 3, and 4), involvement of the corpus callosum with restricted diffusion (Figures 1 and 3), and areas of necrosis (Figure 2). These MRI findings led to the diagnosis of DNL and that was further supported by the exhaustive investigation of other alternative causes. Lumbar puncture was not obtained; however meningitis and encephalitis were not relevant differential diagnosis in the absence of meningeal signs and fever and with a progressive reduction of inflammatory biomarkers.

In the following days the state of coma persisted and the patient eventually died as a consequence of persistent neurological dysfunction. Pathological necropsy studies were considered unnecessary by the combined consideration of MRI findings typical of DNL and thorough exclusion of an alternative diagnosis.

## 3. Discussion

DNL in immunocompetent patients has increasingly been recognized and the diagnosis is challenging not only because of its rarity but also as a result of the nonspecificity of the clinical manifestations. Moreover, both the immunocompromised and the septic shock patients usually have parallel confounding factors which contribute do decreased level of consciousness, particularly intravenous sedatives, and analgesics [8]. The identification of the typical MRI pattern was determinant in our case, in view of the importance and specificity of these findings documented in the medical literature [3, 13]. Still, in many cases, particularly in critically ill patients, the diagnosis is established postmortem after necropsy [1, 2, 6, 8].

The pathobiology of DNL remains unclear [1]. In regard to the association between DNL and septic shock, Sharshar et al. found evidence of higher levels of systemic TNF-*α*, IL-1*β*, IL-6, IL-8, and IL-10, at days one, three, four, and eight of hospitalization in one case, in comparison to two septic shock controls [1]. Despite their small study population, they postulated a possible correlation between those values and DNL [1]. Interestingly, IL-1*β* and TNF-*α* have been proposed to have a role in axonal damage, mediation of neurotoxic factors, regulation of nitric oxide synthase, and apoptotic neuronal death. IL-1*β* can also augment vascular permeability causing brain oedema in other clinical settings [1, 14–20].

There are two reported cases in patients with septic shock with no evidence of previous immunodeficiency [1, 8] which were diagnosed postmortem in contrast to our case where the MRI findings together with an exhaustive exclusion of other causes allowed an antemortem diagnosis.

The relationship between DNL and septic shock raises additional questions, namely, in the interpretation and classification of the septic encephalopathy (SE) syndrome. SE is a common finding with a variable range of clinical manifestations that can be present in about one-third of the patients with septic shock [8]. It is considered a brain or autonomic dysfunction complicating septic shock and the pathogenic mechanism can involve septic embolism in central nervous system, endotoxinemia, immunologic deregulation, vascular ischemic or hemorrhagic complications, metabolic disturbances, and leukoencephalopathy such as posterior reversible leukoencephalopathy [8]. Thus, it is possible to look at DNL as one of the possible causes of SE, rather than an alternative diagnosis, and increased awareness about this relationship will probably identify additional cases where DNL is the primary brain change in patients who would otherwise be diagnosed as having unclassified SE.

In conclusion, we present a case of a patient with septic shock without previous immunodeficiency with severe and fatal neurologic dysfunction, in which a DNL diagnosis was established antemortem on the basis of the typical MRI findings, and after the thorough exclusion of an alternative diagnosis.
